# RCC2 over-expression in tumor cells alters apoptosis and drug sensitivity by regulating Rac1 activation

**DOI:** 10.1186/s12885-017-3908-y

**Published:** 2018-01-10

**Authors:** Nan Wu, Dong Ren, Su Li, Wenli Ma, Shaoyan Hu, Yan Jin, Sheng Xiao

**Affiliations:** 1 Department of Pathology, Brigham and Women’s Hospital, Harvard Medical School, Boston, MA 02115 USA; 2 Department of Medical Genetics, Harbin Medical University, Harbin, China; 3 Children’s Hospital of Soochow University, Suzhou, China; 4 Key Laboratory of Cardiovascular Medicine Research, Harbin Medical University, Ministry of Education, Harbin, China

**Keywords:** RCC2, Rac1, Apoptosis

## Abstract

**Background:**

Small GTP binding protein Rac1 is a component of NADPH oxidases and is essential for superoxide-induced cell death. Rac1 is activated by guanine nucleotide exchange factors (GEFs), and this activation can be blocked by regulator of chromosome condensation 2 (RCC2), which binds the switch regions of Rac1 to prevent access from GEFs.

**Methods:**

Three cancer cell lines with up- or down-regulation of RCC2 were used to evaluate cell proliferation, apoptosis, Rac1 signaling and sensitivity to a group of nine chemotherapeutic drugs. RCC2 expression in lung cancer and ovarian cancer were studied using immunochemistry stain of tumor tissue arrays.

**Results:**

Forced RCC2 expression in tumor cells blocked spontaneous- or Staurosporine (STS)-induced apoptosis. In contrast, RCC2 knock down in these cells resulted in increased apoptosis to STS treatment. The protective activity of RCC2 on apoptosis was revoked by a constitutively activated Rac1, confirming a role of RCC2 in apoptosis by regulating Rac1. In an immunohistochemistry evaluation of tissue microarray, RCC2 was over-expressed in 88.3% of primary lung cancer and 65.2% of ovarian cancer as compared to non-neoplastic lung and ovarian tissues, respectively. Because chemotherapeutic drugs can kill tumor cells by activating Rac1/JNK pathway, we suspect that tumors with RCC2 overexpression would be more resistant to these drugs. Tumor cells with forced RCC2 expression indeed had significant difference in drug sensitivity compared to parental cells using a panel of common chemotherapeutic drugs.

**Conclusions:**

RCC2 regulates apoptosis by blocking Rac1 signaling. RCC2 expression in tumor can be a useful marker for predicting chemotherapeutic response.

## Background

RCC2 was first discovered as a telophase disk-binding protein (TD-60) [1], suggesting its role in mitosis. RCC2 shares significant similarities in primary sequence with RCC1, a known guanine nucleotide exchange factor (GEF) for Ran (ras-related nuclear protein). RCC2, however, failed to interact with Ran, and instead bound Rac1 [2]. RCC2 bound the Rac1 switch regions to block Rac1 GEF access, leading to the attenuation of Rac1 activation [3]. Cells with deficient RCC2 had increased Rac1 activity, which was associated with increased cell adhesion and cell attachment [4]. Rac1 belongs to the Rho family of GTPases, small G-proteins best known for their roles in cytoskeleton rearrangement [5]. Rac1 has, however, also been implicated in superoxide-induced cell death. Rac1 signaling is involved in the generation of reactive oxygen species (ROS) and the expression of activated Rac1 in fibroblasts and HeLa cells results in a significant increase in intracellular ROS [6, 7]. ROS can be generated by various enzymes including NADPH oxidases (Nox). Rac1 is a major activator of Nox 1, 2 and 3; for example, Rac1 can bind both Nox1 and its regulatory subunits NOXA1 to regulate ROS production [8–12]. In mouse fibroblasts, tumor necrosis factor (TNF) induced the formation of a signaling complex including Nox1 and Rac1. Rac1 knock down results in marked decrease in both superoxide generation and superoxide -induced cell death [9].

We found RCC2 over-expression in majority of lung cancer and ovarian cancer in this study. Further studies showed that RCC2 over-expression in tumor cells led to attenuation of spontaneous- or STS-induced apoptosis and the apoptosis resistance was associated with decreased Rac1 activation. An in vitro cell assay showed that various tumor cell lines with RCC2 over-expression were resistant to most chemotherapeutic drugs. These results found a novel role of RCC2 in apoptosis via its interaction with Rac1, and the RCC2 expression level in tumors may be useful in predicting patients’ response to chemotherapy.

## Methods

### Constructs

RCC2 cDNA coding sequence (NM_001136204.2) was fused with N-terminal eYFP (GeneCopoeia clone# EX-E0423-M15; Rockville, MD, USA). The constitutively activated pRK5-myc-Rac1-Q61L was created by Dr. Hall’s lab [13], and the leucine substitution prevents endogenous and GAP-stimulated GTPase activity of Rac1. Plasmid DNAs were prepared with EndoFree® Plasmid kit (Qiagen, Valencia, CA, USA).

### RCC2 siRNAs

Three RCC2-specific siRNAs were used: siG151029043959 (5’-CCACGAAGTGATTGTGTCT), siG151029044022 (5’-GGAGGTAAAGACTCTGGAT) and siG151029044006 (5′- GCCTGTACCAAACGTGGTT). Ribobio Negative Control siRNA was used as negative control (Ribobio Co. Guangzhou, China).

### Transfection

Plasmids or siRNA were transiently transfected into HeLa cells (ATCC® CCL-2™, American Type Culture Collection (ATCC), Manassas, VA, USA), CRL5800 (ATCC® CRL-5800™, ATCC) and MDA-MB-231 (ATCC® HTB-26™, ATCC) using Lipofectamine 3000 (Invitrogen, Grand Island, NY, USA). Stable cell lines expressing YFP or RCC2-YFP were also established by selections with G418 (400 μg/ml) for 3 weeks, and the YFP and RCC2-YFP expression were monitored using an inverted fluorescence microscope.

### Cell proliferation by trypan blue exclusion and soft agar assay

HeLa cells with stable expression of YFP, RCC2-YFP and parental cells were cultured in 96-well plates. Cell counts were determined daily using trypan blue exclusion method. For soft agar assays, 1 × 10^4^ cells were suspended in 2 ml of soft agar (0.35% Bactoagar in DMEM/F12 with 20% FCS), plated onto 5 ml of solidified agar (0.75% Bactoagar in DMEM/F12) in a 6-well plate, and cultured at 37 °C in 5% CO2 for 10 days. Colonies were fixed with methanol and stained with Giemsa.

### Caspase-Glo® 3/7 assay

HeLa cells were transfected with YFP, RCC2-YFP, and/or Rac1-Q61L for 48 h in 96-well plates. Caspase-Glo® 3/7 Reagent (Promega, Madison, WI, USA) was added to cells at a 1:1 ratio (volume), mixed and luminescence measured in a plate-reading luminometer. Results were averaged between six wells from two separate transfections.

### Co-immunoprecipitation

HeLa cells expressing YFP or RCC2-YFP were lysed in 300 μL of lysis buffer and pre-cleared by incubating with 20 μL of protein A–Sepharose (Pharmacia, Piscataway, NJ, USA) for 1 h at 4 °C with gentle rotation. Pre-cleared protein lysate were incubated with rabbit anti-GFP antibody (ab6556, Abcam, Cambridge, MA, USA) overnight at 4 °C and followed by incubating with 10 μl of protein A–Sepharose for 1 h. After three washes in lysis buffer, proteins were eluted at 90 °C in 30 μl of SDS–PAGE loading buffer and resolved by SDS–PAGE under reducing conditions (4%–12% gradient gels). For Western blot analyses, proteins were electrophoretically transferred to polyvinylidene difluoride membranes (Millipore, Waltham, MA, USA), blocked in PBS containing 0.1% Tween 20 (PBST) and 5% dried milk for 1 h, and detected with monoclonal anti-GFP (ab1218, Abcam), anti-Rac1 (ARC03, Cytoskeleton, Denver, CO, USA), anti-cdc42 (ACD03, Cytoskeleton), or anti-Rho A (ARH04, Cytoskeleton) using a chemiluminescence method (ECL; Amersham, Piscataway, NJ, USA).

### Rho GTPases -pull down assay

Rho GTPase pull down was performed with a RhoA/Rac1/Cdc42 Activation Assay Combo Biochem Kit™ (Cytoskeleton; Denver, CO, USA). Briefly, HeLa cells expressing YFP or RCC2-YFP were cultured in serum-free medium overnight and stimulated by adding 1.3 ml FBS per 5 ml medium for 5 min. Cells were washed with cold PBS and lysed in cold cell lysis buffer with a cell scraper. 600 μg of protein lysate were incubated with 25 μl rhotekin-RBD or PAK-PBD beads at 4 °C for 1 h. The beads were washed once with wash buffer and beads-binding proteins eluted in loading buffer and Western blotted with antibodies to RhoA, Rac1 or Cdc42. For total Rho GTPase, crude protein lyses without pull down were evaluated.

### Evaluation of drug sensitivity

HeLa cells, CRL5800 and MDA-MB-231 expressing YFP or RCC2-YFP were cultured in 96-well plates, treated with vehicles or increasing doses of chemotherapeutic drugs for 48 h, and live cells quantitated by CellTiter-Glo® Luminescent Cell Viability Assay (Promega). Vehicles were 0.9% NaCl (Cisplatin) or DMSO (Taxol, Nocodazole, hydroxyurea, Daunorubicin, CPT, STS, 5-Fluorouracil and Irinotecan) and drugs were dissolved in vehicles at 1,000X stock concentration. The surviving cells were calculated as the fraction of vehicle controls. Results were averaged between six wells per dose in two experiments.

### RCC2 expression in tumor tissue microarray

Lung carcinoma progression tissue microarray (LC2083; Biomax; Rockville, MD, USA) and ovarian cancer and normal tissue high density tissue microarray (OV208; Biomax) were de-paraffinized in xylene, antigen-retrieved by heating in 0.01 M sodium citrate buffer (pH 6.0) at 95 °C for 10 min, blocked in 10% normal goat serum for 30 min and incubated with an anti-RCC2 antibody (D14F3; Cell Signaling, Danvers, MA, USA) overnight at 4 °C. Immunohistochemistry staining was performed using a mouse and rabbit specific HRP/AEC (ABC) detection IHC kit (Abcam Ab93705; Boston, MA, USA). RCC2 expression was scored by two experienced researchers. Cases with inconsistent scoring were reviewed by a third pathologist.

### Statistical analysis

All statistical analysis was performed using SPSS 21.0 (IBM, Armonk, NY, USA). The differential expression level of RCC2 between cancers and normal tissues was evaluated by the Mann-Whitney U test. The correlation between RCC2 expression and clinicopathologic features of patients with lung or ovarian cancers was analyzed by the two-tailed χ2 test. All the data was analyzed after excluding the cases with missing values. Other data was expressed as the mean ± standard deviation. One-Way ANOVA multiple comparisons and Bonferroni correction were used to analyze the statistical significance between multiple groups. *P* < 0.05 was considered statistically significant.

## Results

### RCC2-YFP expression attenuated apoptosis

**Fig. 1:**
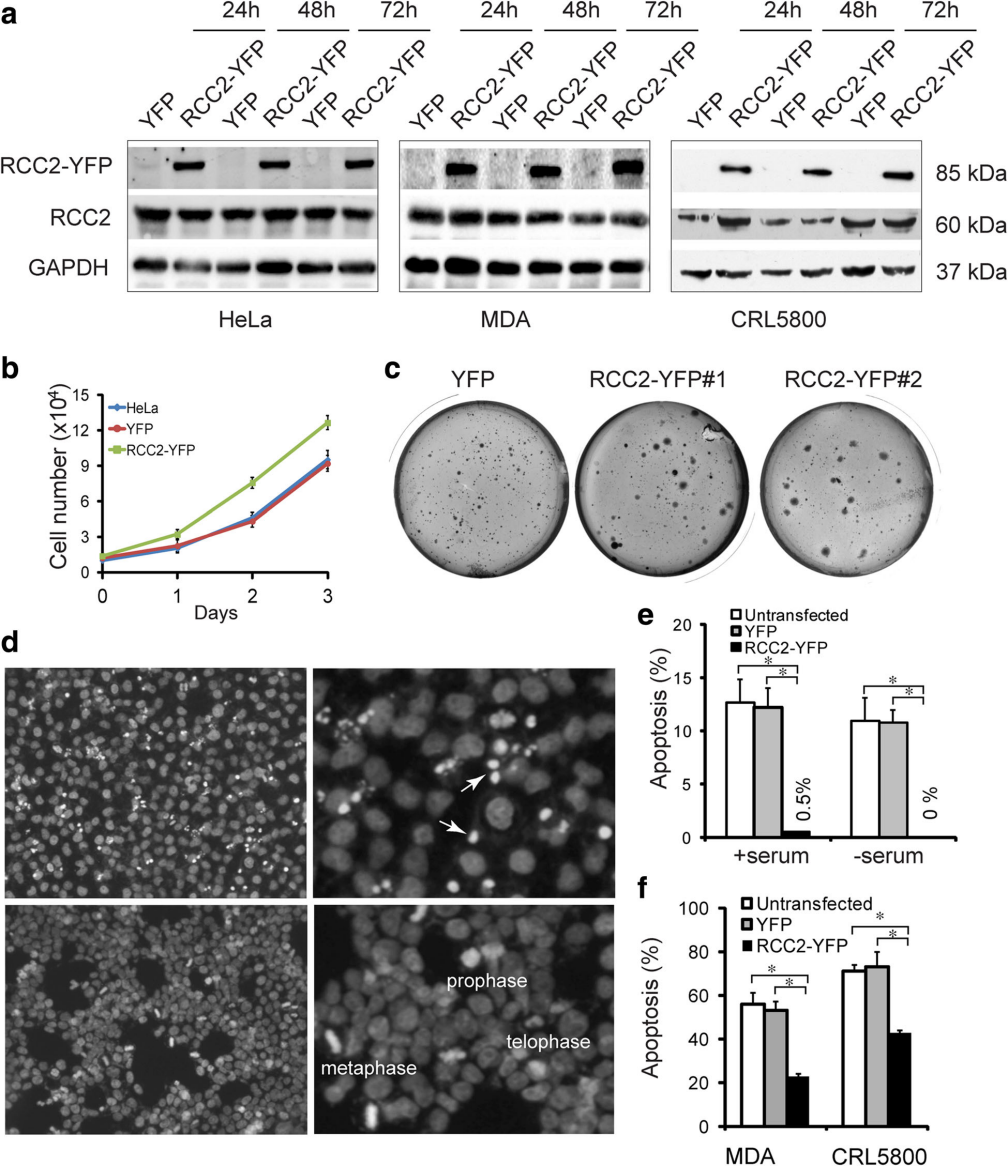
Tumor cells expressing RCC2-YFP are resistant to apoptosis. **a** Tumor cell lines HeLa, MDA-MB-231 and CRL5800 were transfected with a RCC2-YFP expression plasmid or a control YFP plasmid for 24 h, 48 h and 72 h. Western blot analysis with a RCC2-specific antibody showed both RCC2-YFP and endogenous RCC2 in these cells. **b** HeLa cells were cultured in 96 well plates for 1–3 days and counted by trypan blue stain. Cells expressing RCC2-YFP had increased cell proliferation compared to control YFP HeLa cells or parental cells (mean ± S.D. of four replicates, *P* < 0.05 at day 3). **c** HeLa cells were cultured in soft agar plates for 10 days. RCC2-YFP #1 and #2 were cells from two independent transfections. Bigger cell colonies were seen in the RCC2-YFP-expressing cells as compared to the YFP-expressing cells. **d** & **e** HeLa cells were transiently transfected with YFP or RCC2-YFP for 24 h, fixed and counterstained with DAPI. Apoptotic cells were scored by nucleus pyknosis and fragmentation (arrows). RCC2-YFP expression virtually eliminated spontaneous apoptosis in both serum-free and 10% FBS culture conditions (mean ± S.D. of four replicates, *P < 0.01) (**e**). **f** MDA-MB-231 and CRL5800 cells were transiently transfected with YFP or RCC2-YFP, treated with 10 μM STS for 48 h and apoptotic cells scored by DAPI stain. RCC2-YFP expression partially blocked the STS-induced apoptosis (mean ± S.D. of four replicates, *P < 0.01)

RCC2-YFP was transiently expressed in three tumor cell lines including HeLa, lung cancer CRL5800 and breast cancer MDA-MB-231. Approximately 80% of CRL5800 or MDA-MB-231 cells and 90–100% of HeLa cells were YFP-positive 24 h after transfection. RCC2-YFP was localized at nuclei in interphase cells and midbody/midzone region in anaphase cells, similar to those endogenous RCC2 [2]. RCC2-YFP expression was confirmed by a western blot analysis with an anti-RCC1 antibody (Fig. 1a). The RCC2-YFP expression resulted in significant increase in HeLa cell proliferation, but not in CRL5800 and MDA-MB-231 (Fig. 1b). Increased cell growth was also confirmed in RCC2-YFP HeLa cells seeded in soft agar plates (Fig. 1c). By DAPI stain, approximately 10~15% of HeLa cells were spontaneously apoptotic cultured in medium containing either 10% FBS or serum-free medium. By contrast, virtually no apoptotic cells were detected in these cells expressing RCC2-YFP (Fig. 1d & e), suggesting that RCC2-YFP expression blocked the spontaneous apoptosis of HeLa cells. Both CRL5800 and MDA-MB-231 cells showed no spontaneous apoptosis in culture. To study whether the RCC2-YFP expression also confers protection against apoptosis in these cells, STS (10 μM) was added to the cultured cells for 48 h and apoptosis was scored by DAPI stain. Both cell lines with RCC2-YFP expression were significantly more resistant to STS-induced cell death when compared to control cells (Fig. 1f).

**Fig. 2:**
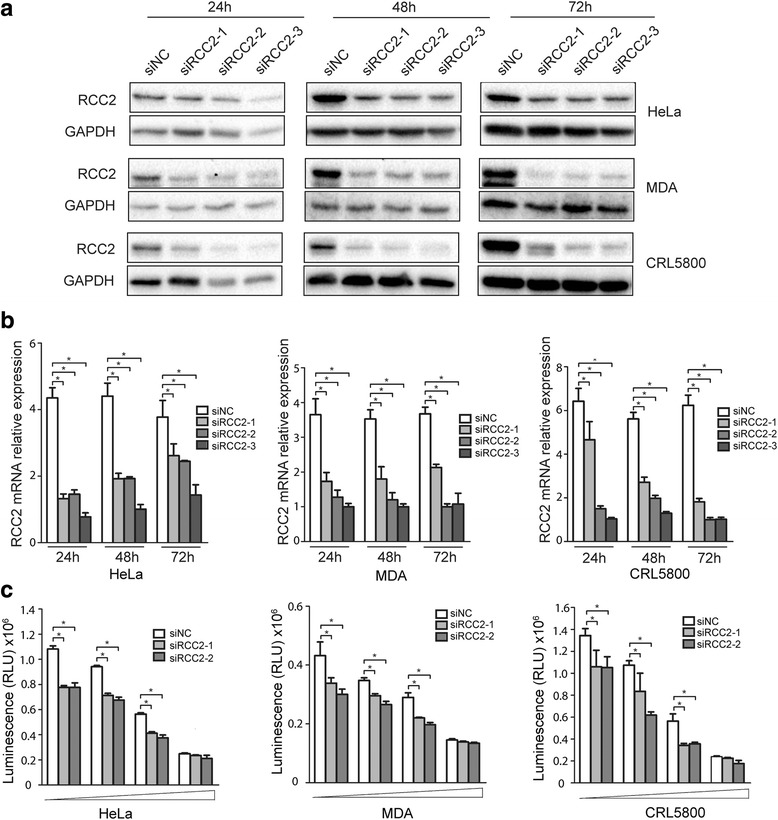
RCC2 down regulation in tumor cells led to increased sensitivity to STS treatment. **a** & **b** Tumor cell lines HeLa, MDA-MB-231 and CRL5800 were transfected with 3 RCC2-specific siRNA oligos (siRNA-RCC2–1, siRNA-RCC2–2, siRNA-RCC2–3) or a control siRNA (siNC) for 24 h, 48 h, and 72 h. RCC2 protein (**a**) and mRNA (**b**) were evaluated by Western blotting and quantitative real-time RT-PCR respectively. All 3 RCC2-specific siRNAs were effective in reducing RCC2 expression (mean ± S.D. of four replicates, *P < 0.01). **c** Tumor cells transfected with siRNA-RCC2–1, siRNA-RCC2–2, or siNC for 24 h were treated with STS at increasing concentrations (0.05, 0.1, 0.2 and 0.4 μM for HeLa; 5, 8, 10, 20 μM for MDA-MB-231; 5, 10, 30 and 50 μM for CRL5800). Surviving cells were estimated by a CellTiter-Glo® Luminescent Cell Viability Assay. Luminescence values represent the mean ± S.D. of six wells, *P < 0.05)

All three cell lines, HeLa, CRL5800 and MDA-MB-231, expressed endogenous RCC2 (Fig. 1a). We studied whether a decreased RCC2 in these cells will alter their sensitivity to STS-induced apoptosis. Expression of three sets of RCC-specific siRNA for 24 h, 48 h and 72 h all resulted in significant RCC2 knock down at both mRNA and protein level (Fig. 2a and b). These cells were treated with increasing STS for 24 h and surviving cells were quantitated using a CellTiter-Glo® Luminescent Cell Viability Assay. As shown in Fig. 2c, cells expressing RCC2-specific siRNA were consistently more sensitive to STS at different concentration as compared to the control cells expression a control siRNA. At the highest STS concentration, most cells were killed with or without RCC2 knock down.

### RCC2 interrupts apoptosis by blocking Rac1 activation

**Fig. 3:**
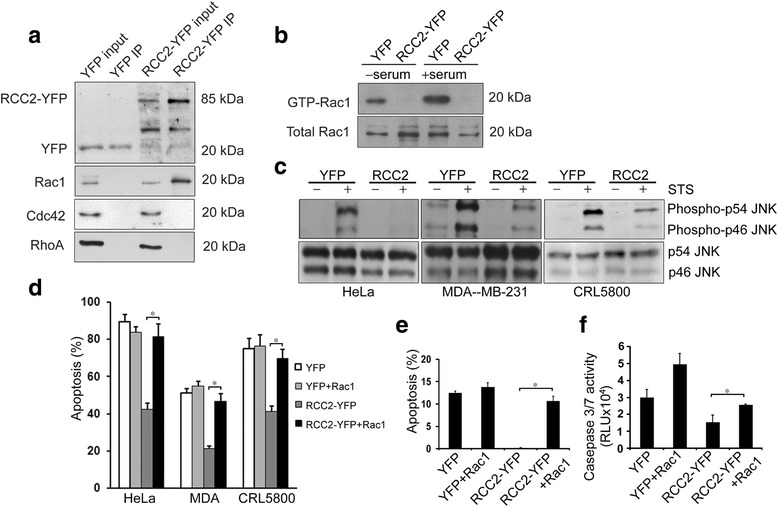
RCC2-YFP interrupts apoptosis via blocking Rac1 activation. **a** Protein lysate from HeLa cells expressing YFP or RCC2-YFP were immunoprecipitated with anti-GFP antibody and Western blotted with anti-GFP, anti-Rac1, anti-cdc42, or anti-RhoA antibody. Rac1 was co-precipitated with RCC2-YFP. **b** HeLa cells expressing YFP or RCC2-YFP were serum-starve1d overnight followed by serum-stimulation for 5 min. GTP-bound activated Rac1 was pulled down by PAK-PBD beads and Western blotted with anti-Rac1 antibody. RCC2-YFP expression blocked both endogenous and serum-induced Rac1 activation. **c** HeLa, MDA-MA-231 and CRL5800 cells expressing YFP or RCC2-YFP were treated with STS (0.5 μM for HeLa; 10 μM for MDA and CRL5800) for 30 min and Western blotted with antibodies to phospho-JNK and total JNK. RCC2-YFP expression blocked the STS-induced JNK phosphorylation in all three cell lines. **d** Tumor cells transfected with Rac1-Q61L, RCC2-YFP or both for 48 h were treated with STS (0.5 μM for HeLa; 10 μM for MDA and CRL5800) and apoptotic cells were scored by DAPI stain after 24 h treatment. Rac1-Q61L expression in tumor cells revoked the protection of RCC2-YFP towards STS-induced apoptosis (mean ± S.D. of four replicates, *P < 0.05). **e** HeLa cells transfected with Rac1-Q61L, RCC2-YFP or both for 48 h in the presence of serum. Spontaneous apoptotic cells were scored by DAPI stain. Rac1-Q61L expression in HeLa cells revoked the protection of RCC2-YFP toward spontaneous apoptosis (mean ± S.D. of four replicates, *P < 0.01). **f** HeLa cells were transfected with Rac1-Q61L, RCC2-YFP or both in the presence of serum, and Caspase-Glo® 3/7 assay was performed 48 h after transfection. Rac1-Q61L and RCC2-YFP expression in HeLa cells affected Caspase 3/7 activity (mean ± S.D. of six wells, *P < 0.05)

RCC2 is capable of directly binding Rac1 [2]. Because multiple studies suggested a role of Rac1 in apoptosis, we explored the possibility that RCC2-YFP attenuates apoptosis by blocking Rac1 activation. We first evaluated the interaction between RCC2-YFP and endogenous Rac1 in HeLa cells expressing YFP or RCC2-YFP. A co-immunoprecipitation assay was performed, which confirmed the presence of Rac1, but not of Rho A or cdc42, in the RCC2-YFP pull down complex (Fig. 3a). We next evaluated the effectiveness of RCC2-YFP expression on blocking the serum-induced Rac1 activation. HeLa cells expressing YFP or RCC2-YFP were serum-starved for 48 h, followed by 20% serum stimulation for 5 min. Activated Rac1 was pulled down by GST-PAK-p21 binding domain (PBD). Serum stimulation led to increased Rac1 activation in YFP cells as expected; however, Rac1 activation was not detected in RCC2-YFP HeLa cells, confirming that the RCC2-YFP expression blocked the serum-stimulated Rac1 activation (Fig. 3b). In addition, YFP HeLa cells had endogenous Rac1 activation in serum-free culture, which was also blocked by the expression of RCC2-YFP (Fig. 3b). Similar pull down assays showed no significant difference for other Rho GTPases including RhoA and cdc42 (data not shown). Rac1 activation leads to C-Jun kinase (JNK) activation, i.e.*,* its phosphorylation at Thr183 and Tyr185. Western blot analysis showed that the RCC2-YFP expression blocked the STS-induced JNK phosphorylation in all three cell lines, consistent with Rac1 inactivation (Fig. 3c). We then co-transfected tumor cells with both RCC2-YFP and a constitutively activated Rac1-Q61L. Apoptosis was induced by adding STS and scored by DAPI stain. The Rac1-Q61L expression largely revoked the apoptosis protection by RCC2-YFP in these cells (Fig. 3d). In addition, the co-expression of Rac1-Q61L neutralized the protection of RCC2-YFP against spontaneous apoptosis in HeLa cells (Fig. 3e). By a Caspase-Glo® 3/7 assay, RCC2-YFP HeLa cells had significantly decreased activity of Caspase 3/7 as compared to control cells (Fig. 3f).

### RCC2 expression and drug sensitivity

**Fig. 4:**
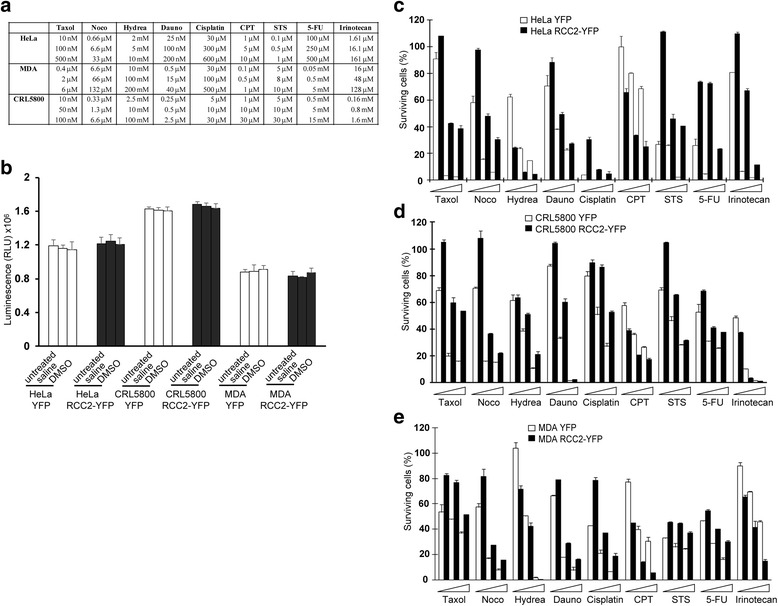
RCC2-YFP expression in tumor cells affects drug sensitivity. **a** HeLa, CRL5800 and MDA-MA-231 cells expressing YFP or RCC2-YFP were treated with vehicles or increasing doses of Taxol, Nocodazole (Noco), Hydroxyurea (Hydrea), Daunorubicin (Dauno), Cisplatin, Camptothecin (CPT), Staurosporine (STS), 5-Fluorouracil (5-FU) or Irinotecan for 48 h. **b, c, d** and **e** Cells were quantitated by a CellTiter-Glo® Luminescent Cell Viability Assay. No significant difference was observed between untreated cells and vehicle-treated cells (0.1% DMSO or 0.1% saline) (**b**). Surviving cells were calculated as the fraction of vehicle controls (**c, d** and **e**). Results were averaged between six wells per dose in two experiments (mean ± S.D.)

The apoptotic mechanisms for chemotherapeutic drugs are often cell- and/or drug-specific; and Rac1/JNK pathways are critical to drug-induced cell death in several settings [14–16]. We established a series of cell lines stably expressing YFP or RCC2-YFP. These cells were evaluated for their sensitivity to a panel of nine common chemotherapeutic drugs (Fig. 4a). Tumor cells were treated with drugs or vehicles for 48 h and cells quantitated by a CellTiter-Glo® Luminescent Cell Viability Assay. No significant difference was observed between untreated cells and vehicle-treated cells (0.1% DMSO or 0.1% saline) (Fig. 4b). All cell lines expressing RCC2-YFP were more resistant to Taxol, Nocodazole, Daunorubicin, Cisplatin, STS and 5-Fluorouracil (5-FU) when compared to control YFP cells; however, these cells were more sensitive to Camptothecin (CPT), a DNA topoisomerase I inhibitor. The response to Irinotecan and Hydroxyurea was cell line-specific: the expression of RCC2-YFP in both CRL5800 and MDA-MB-231 cells resulted in increased sensitivity to Irinotecan; however, RCC2-YFP HeLa cells showed decreased sensitivity to Irinotecan. The RCC2-YFP expression in MDA-MB-231 and HeLa cells resulted in increased sensitivity to Hydroxyurea, although RCC2-YFP CRL5800 cells showed decreased sensitivity to Hydroxyurea (Fig. 4c, d & e).

### RCC2 over-expression in tumor

**Fig. 5:**
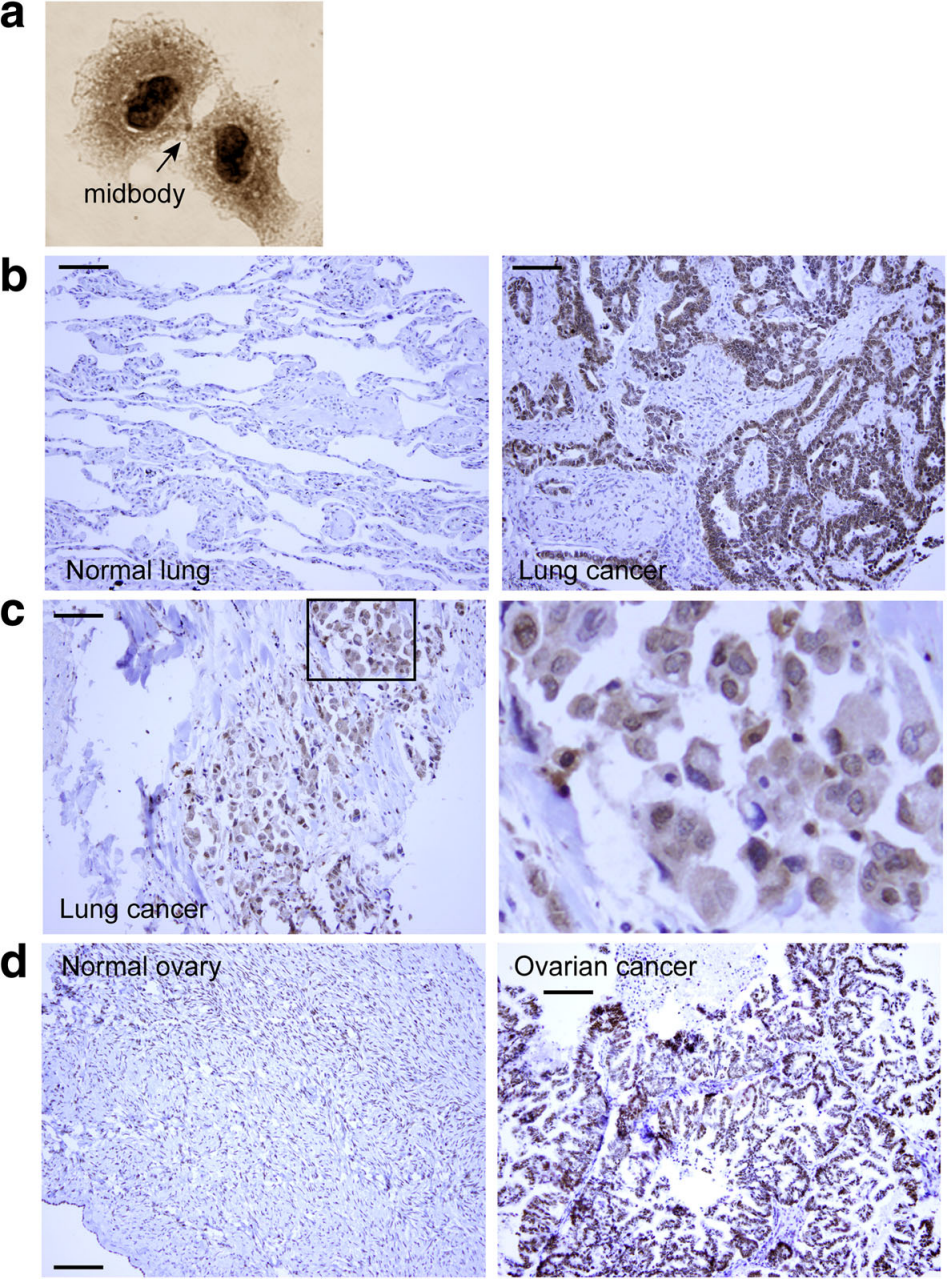
RCC2 was over-expressed in lung cancers and ovarian cancers. **a** IHC of HeLa cells was performed with anti-RCC2 antibody (D14F3, cell signaling). RCC2 signals were seen in nucleus and midbody of a telophase cell as expected. Cytoplasmic RCC2 was also observed. **b** RCC2 expression in a lung cancer tissue microarray was evaluated by IHC. RCC2 were not seen in normal lung (left) but highly expressed in lung cancer (10×, scale bar: 100 μm). In addition to nuclei, RCC2 signals were also seen in cytoplasm in some lung cancers (**c**). **d** RCC2 expression in an ovarian cancer tissue microarray was evaluated by IHC. Normal ovaries expressed none or weak RCC2 (−~+) (left). Increased RCC2 expression was seen in majority of ovarian cancers

RCC2 expression in lung cancer and ovarian cancer was evaluated by IHC of tissue microarrays. The anti-RCC2 antibody (D14F3, cell signaling) was validated by IHC of cultured HeLa cells, which showed a nuclear localization in interphase cells and spindle midzone/midbody localization in mitosis as expected (Fig. 5a). RCC2 expression on tumor tissue microarrays was studied using a 5-tier scoring system (−, −/+, +, ++, +++) depending on signal intensity. In 120 cases of human lung cancers including various types (LC2083; Biomax), 106 (88.3%) had RCC2 expression at +~+++, and 14 of them had - or −/+ (11.7%). In contrast, none of 23 normal lungs expressed RCC2 (Fig. 5b). Cancer-adjacent normal lung tissues and inflammatory pseudotumors expressed low-level RCC2.

(− or−/+)(*P* < 0.001). RCC2 was mainly localized in the nuclei of tumor cells, although some had significant cytoplasmic RCC2 in addition to nuclear localization (Fig. 5c). RCC2 expression level was not significantly different among three grades of non-small cell lung cancer (well-, moderately- and poorly differentiated tumors). Detailed clinicopathological characteristics of lung cancers and RCC2 expression level were listed in Table 1. In an ovarian cancer tissue array (OV208), increased RCC2 expression (++ ~ +++) was detected in 92 of 141 cases of ovarian cancers (65.2%); and 62 of them had RCC2 at +++ (44%). Most normal ovaries expressed none or weak RCC2 (−, −/+ or +)(Fig. 5d). RCC2 expression was significantly higher in metastatic ovarian cancers (*P* < 0.05), higher grade tumors (*P* < 0.05) and bigger tumor (*P* < 0.05). Detailed clinicopathological characteristics of ovarian cancers and RCC2 expression level were listed in Table 2.

**Table 1:** Correlation between RCC2 expression and clinicopathological features in lung cancer

Variables	Number	RCC2 expression				*P* value
		Low expression (−~−/+)		High expression (+~+++)		
Gender						
Female	50	15	(30.0%)	35	(70.0%)	0.262^a^
Male	152	59	(38.8%)	93	(61.2%)	
Age						
≤ 56	88	34	(38.6%)	54	(61.4%)	0.604^a^
> 56	114	40	(35.1%)	74	(64.9%)	
Type						
Non-malignant^c^	52	45	(86.5%)	7	(13.5%)	0.000^a^
Malignant	120	14	(11.7%)	106	(88.3%)	
Metastatic	30	15	(50.0%)	15	(50.0%)	
Tissue						
Lung	120	14	(11.7%)	106	(88.3%)	0.000^a^
Lymph node	30	15	(50.0%)	15	(50.0%)	
Pathology						
Non-malignant	60	45	(75.0%)	15	(25.0%)	0.000^b^
Adenocarcinoma	41	7	(17.1%)	34	(82.9%)	
Squamous cell	40	8	(20.0%)	32	(80.0%)	
Large cell	6	2	(33.3%)	4	(66.7%)	
Others^d^	50	8	(16.0%)	42	(84.0%)	
Grade						
1	27	4	(14.8%)	23	(85.2%)	0.278^a^
2	19	2	(10.5%)	17	(89.5%)	
3	25	7	(28.0%)	18	(72.0%)	

“a” detected by Pearson’s χ^2^ tests, “b” detected by continuity corrected χ^2^ test. “c” non-malignant include 23 cases of normal lung, 19 cases of inflammation and 10 cases of benign tumor. “d” Others include 21 cases of small cell lung cancer 24 cases of bronchioloalveolar carcinoma and five cases of carcinoma sarcomatodes. Grade is only for non-small cell lung cancer

**Table 2:** Correlation between RCC2 expression and clinicopathological features in ovarian cancer

Variables	Number	RCC2 expression				*P* value
		Low expression (−~+)		High expression (++~+++)		
Age						
≤ 45	83	47	(56.6%)	36	(43.4%)	0.000^a^
> 45	117	31	(26.5%)	86	(73.5%)	
Type						
Normal	23	22	(95.7%)	1	(4.3%)	0.000^b^
Malignant	141	49	(34.8%)	92	(65.2%)	
Metastasis	36	7	(19.4%)	29	(80.6%)	
Tissue						
Ovarian	141	49	(34.8%)	92	(65.2%)	0.078^a^
Others^c^	36	7	(19.4%)	29	(80.6%)	
Pathology						
Normal	23	22	(95.7%)	1	(4.3%)	0.000^b^
Serous	69	8	(11.6%)	61	(88.4%)	
Mucinous	46	35	(76.1%)	11	(23.9%)	
Serous papillary	36	6	(16.7%)	30	(83.3%)	
Mucinous papillary	23	7	(30.4%)	16	(69.6%)	
Grade						
1	59	33	(55.9%)	26	(44.1%)	0.000^b^
2	58	11	(19.0%)	47	(81.0%)	
3	42	1	(2.4%)	41	(97.6%)	
TNM stage						
T						
1	70	29	(41.4%)	41	(58.6%)	0.031^a^
2–4	63	15	(23.8%)	48	(76.2%)	
N						
0	60	22	(36.7%)	38	(63.3%)	0.542^a^
1	36	11	(30.6%)	25	(69.4%)	
M						
0	123	43	(35.0%)	80	(65.0%)	0.707^b^
1	12	3	(25.0%)	9	(75.0%)	

“a” detected by Pearson’s χ^2^ tests, “b” detected by continuity corrected χ^2^ test. “c” others include three cases of lymph node, three cases of abdominal wall, nine cases of epiploon, three cases of groin, three cases of mesentery, nine cases of rectum, three cases of spleen, and three cases of vermiform appendix

## Discussion

Two separate genome-wide screenings found a possible role of RCC2 in tumorigenesis. By genotyping 930 patients with cutaneous basal cell carcinoma (BCC) and 33,117 controls, a single nucleotide polymorphism (SNP) rs7538876, which is located in the vicinity of RCC2, was associated with increased risk of BCC by 2.98 times as compared to non-carriers [16]. Similar studies on 891 prospectively accrued melanoma patients showed that the same rs7538876 was associated with early recurrence of melanoma by an average of 2 years [17]. Further studies found that the rs7538876 variant is involved in RCC2 promoter CpG methylation and is associated with increased RCC2 expression [17]. RCC2 is also a downstream target of the known cancer related miR-29c through its 3′ untranslated region (3′ UTR) miR-29c target sequence, and RCC2 expression is negatively regulated by miR-29c. In advanced gastric cancer, miR-29c was significantly down-regulated, leading to RCC2 over-expression, and the expression of the RCC2-specific siRNA in these cells resulted in increased cell death and decreased proliferation [18]. A recent study found RCC2 as a target gene for DNA mismatch repair (MMR) deficiency in colon cancer. MMR deficiency leads to DNA microsatellite instability. RCC2 has a mononucleotide (A)_10_-repeat within the 5′ UTR. Deletion of one or two bases in this region is found in colorectal cancer with MMR deficiency, and this deletion is associated with altered mRNA structure, decreased RCC2 expression, and favorable prognosis in colorectal cancer with microsatellite instability, suggestive of an oncogenic role of RCC2. Contradictorily, increased RCC2 expression is associated with favorable prognosis in a subgroup of colorectal cancer with microsatellite stability [19].

In this study, we found that RCC2 plays a role in tumor cell death by blocking the Rac1- initiated apoptosis. Resistance to apoptosis is one of the hallmarks of malignancy, which contributes to both tumorigenesis and tumor progression by allowing damaged cells to escape surveillance mechanisms, leading to accumulation of mutations beneficial to cell transformation and proliferation. Apoptosis is a highly complex and sophisticated process with many modulators. The role of Rac family in apoptosis was first suggested by thymus atrophy in mice expressing activated Rac2, a hematopoiesis-specific Rac family member, consistent with a Rac2-depedent apoptosis pathway in T lymphocytes [20]. Further studies found that Rac1 is a key proapoptotic modulator in a variety of cell types in response to different apoptotic stimuli, including UV-induced apoptosis in Rat-2 fibroblasts [21], β-adrenergic receptor-modulated apoptosis in rat ventricular myocytes [22], growth factor deprivation-induced apoptosis in human hepatoma cells [23], capsaicin-induced apoptosis in human breast epithelial cells [24], TNF-α-induced apoptosis in intestinal epithelial cells ([25], hyperglycemia-induced apoptosis in cardiomyocytes [26], and Taxol-induced apoptosis in human melanoma cells [27]. Paradoxically, Rac1 can also act anti-apoptotically. Examples of these include Cu/Zn-superoxide dismutase (SOD1) mutant-induced motoneuronal cell death [28], Cisplatin-induced apoptosis in NIH3T3 cells [29], UV-induced apoptosis in COS-1 cells [30], TIPE1- induced apoptosis in hepatocellular carcinoma cells [31], and TNF-α-induced apoptosis in endothelial cells [32]. These studies suggest that Rac1 can play either pro-apoptotic or anti-apoptotic roles, depending on cellular context and/or apoptosis inducers.

Because of the importance of Rac1 in apoptosis, and because RCC2 expression effectively blocked Rac1 activation, it is not surprising that tumor cells with forced RCC2 expression reacted differently to drug-induced apoptosis. In the three cancer cell lines tested, forced RCC2 expression led to drug resistance to most chemotherapeutic reagents, although increased sensitivity was also observed in some settings. These results are consistent with the complex roles of Rac1 on apoptosis. We found all three cell lines with RCC2 expression had increased sensitivity to Camptothecin. This is consistent with a report that a Rac1 inhibitor (equivalent of RCC2 overexpression) increased the sensitivity of glioblastoma cell lines to Camptothecin [33]. Currently, nano-particle Camptothecin has been used to treat relapsed/refractory small cell lung cancer and advanced non-small cell lung cancer in clinical trials. It will be interesting to evaluate whether RCC2 expression level in these tumors affects their sensitivity to this drug. We also found that two of three cell lines with RCC2 expression had increased sensitivity to Irinotecan and Hydroxyurea. Irinotecan is a semisynthetic analog of camptothecin, and therefore may have similar anti-cancer mechanisms to camptothecin. Hydroxyurea is capable of inducing Rac1 accumulation in nuclei [34]; and nuclear Rac1 activity increased cell proliferation [35]. Therefore, cells with RCC2 overexpression may have increased sensitive to Hydroxyurea by down-regulating Rac1 activity in these cells.

## Conclusions

In summary, our studies found that RCC2 is often overexpressed in lung cancer and ovarian cancer, and RCC2 expression in cancer cells altered their sensitivity to drug-induced cell death, probably because of its interaction with cellular Rac1 activity. Our results suggest RCC2 as a useful marker for predicting chemotherapeutic response.

## Abbreviations

5-FU: 5-Fluorouracil
CPT: Camptothecin
GEFs: Guanine nucleotide exchange factors
MMR: DNA mismatch repair
Nox: NADPH oxidases
RCC2: Regulator of chromosome condensation 2
ROS: Reactive oxygen species
TNF: Tumor necrosis factor
